# An Endoplasmic Reticulum-Targeted Ratiometric Fluorescent Molecule Reveals Zn^2+^ Micro-Dynamics During Drug-Induced Organelle Ionic Disorder

**DOI:** 10.3389/fphar.2022.927609

**Published:** 2022-06-06

**Authors:** Hongbao Fang, Yaheng Li, Shankun Yao, Shanshan Geng, Yuncong Chen, Zijian Guo, Weijiang He

**Affiliations:** ^1^ State Key Laboratory of Coordination Chemistry, School of Chemistry and Chemical Engineering, Chemistry and Biomedicine Innovation Center (ChemBIC), Nanjing University, Nanjing, China; ^2^ Nanchuang (Jiangsu) Institute of Chemistry and Health, Nanjing, China

**Keywords:** organelle targeting, Zn^2+^ dynamics, ER stress, ionic disorder, drug regulation

## Abstract

The endoplasmic reticulum (ER) is the main storage site of Zn^2+^, and Zn^2+^ plays an important role in regulating ER homeostasis. Therefore, we designed and synthesized a ratiometric fluorescent Zn^2+^ probe ER-Zn targeting ER stress. The probe displayed a specific Zn^2+^ induced blue shift at the spectral maximum values of excitation (80 nm) and emission (30 nm). The ratio imaging capability of Zn^2+^ under dual excitation mode can be applied not only to quantitative and reversible detection of exogenous Zn^2+^, but also the observation of the Zn^2+^ level change under ER stress, elucidating the different behaviors of Zn^2+^ release in ER stimulated by tunicamycin and thapsigargin. Additionally, the NIR imaging capability of ER-Zn provides an important basis for further research on animal models and is expected to realize the visualization and treatment of ER stress-related diseases through the regulation of ER stress by Zn^2+^. We envision that this probe can be applied to screen drugs for diseases related to ER stress regulation.

## 1 Introduction

Zinc (Zn^2+^) is the second most abundant transition metal ion after iron in the body, involving in a variety of pathological and physiological processes, such as enzyme transport, gene transcription, and immune function (Kambe et al., 2015; Maret, 2019; Bird and Wilson, 2020). Endoplasmic reticulum (ER) homeostasis was found to be closely related to Zn^2+^. It has been reported that zinc deficiency can cause ER stress and a decrease of metallothionein level, which leads to oxidative stress, cell damage, and acute kidney injury (Inagi, 2009; Hadj Abdallah et al., 2018; Hancock et al., 2020). Therefore, it is necessary to detect Zn^2+^ level changes in ER and even *in vivo* to study the physiological process associated with ER.

Fluorescent probe method has been widely used to detect ionic dynamics in living cells due to its advantages of low toxicity, easy preparation, and *in situ* non-destructive detection (Chen et al., 2015; Li et al., 2020; Chen et al., 2021; Fang et al., 2021a; Liu et al., 2021; Wang and Diao, 2022; Wang et al., 2022). Therefore, the use of fluorescent probes to study Zn^2+^ in ER provides a powerful tool for the detection and treatment of ER stress-related diseases by regulating the Zn^2+^ level (Chabosseau et al., 2018; Huang et al., 2021; Liu et al., 2022). Although many Zn^2+^ fluorescent probes have been developed, most of them show turn-on response and are difficult to use for *in vivo* detection (Fang et al., 2021b; Qi et al., 2021; Wang et al., 2021).

In this paper, due to the lack of research on near-infrared (NIR) ratiometric Zn^2+^ fluorescent probes targeting ER, such a probe combined with the intramolecular charge transfer (ICT) mechanism was rationally designed by us. For one thing, the styrene group was introduced into the α site of BODIPY fluorophore, which not only increased its excitation wavelength, but also extended the emission wavelength of the fluorophore to the NIR region for reducing the interference of spontaneous fluorescence in organisms and damage to cells during imaging. For another, *N*, *N*, *N*′-tri (pyridin-2-ylmethyl) ethane-1,2-diamine (TPEA) was introduced at the *α* position as the Zn^2+^ chelating group. Finally, p-toluenesulfonamide was introduced at the meso position of the fluorophore as the ER targeting group to construct a highly selective NIR Zn^2+^ fluorescent probe targeting ER, ER-Zn. Cell imaging experiments showed that ER-Zn had good cytocompatibility, which not only realized reversibly ratiometric imaging of exogenous Zn^2+^, but also quantitatively monitored the fluctuation of Zn^2+^ level in ER under ER stress. ER-Zn provides a new method to study the relationship between ER stress regulation and Zn^2+^ level.

## 2 Materials and Methods

### 2.1 Materials and Instruments

The commonly used chemicals (e.g., potassium carbonate, piperazine, acid and base, etc.) and general solvents (e.g., dichloromethane, methanol, ethanol, ethyl acetate, petroleum ether, etc.) were all from the domestic reagents purchased by School of Chemistry and Chemical Engineering of Nanjing University, and they were not further purified before use. In the spectral characterization, the solvents such as DMSO and DMF were both spectrally pure reagents purchased from Aldrich. The water used was ultrapure from Millipore. CuSO_4_, MgCl_2_·6H_2_O, CaCl_2_, Zn(NO_3_)_2_·7H_2_O, NaCl, KCl, FeCl_2_, CoCl_2_·6H_2_O, and NiCl_2_·6H_2_O were dissolved in ultrapure water to prepare corresponding concentrations of metal ion stock solution. The stock solution of Zn^2+^ was prepared by dissolving ZnCl_2_ in ultrapure water.

Mass spectrometry was determined by LCQ electrospray ionization mass spectrometry (ESI-MS, Finnigan). High-resolution mass spectrometry (HRMS) was determined by Thermo scientific quadrupole orbit trap tandem high-resolution mass spectrometer. ^1^H and ^13^C NMR were determined by Bruker DRX-500 and Bruker DRX-400 NMR spectrometers using standard pulse sequence with tetramethylsilane (TMS) as internal standard (298 K). The absorption spectra were measured by PerkinElmer Lambda 35 UV-vis spectrometer. Fluorescence emission spectra were recorded by FluoroMax-4 fluorescence spectrometer (HORIBA Jvon Inc.). The pH assay was determined by SevenCompact S210 pH meter (Mettle Toledo). Cell imaging was performed on Olympus FV10-ASW laser confocal fluorescence microscope and Leica SP8 STED 3X confocal microscope. The excitation wavelength was 570 and 650 nm, and the collection wavelength was 660–720 nm.

### 2.2 Synthesis and Characterization

The synthesis steps of ER-Zn are shown in Supplementary Figure S1. 1 and 2 were synthesized according to the literature method (Aydin et al., 2016), and 3 and ER-Zn were synthesized as described in the literature after corresponding improvement.

At 0°C, 3 (3.3 mmol, 1.12 g) and 4-methylbenzenesulfonyl chloride (3.3 mmol, 627 mg) were dissolved in dichloromethane (20 ml), and then several drops of pyridine were added to the solution. After stirring for 2 h, the solvent was removed by rotary evaporation. The target product in orange solid form (1.34 g, 82.3%) was obtained by using dichloromethane/petroleum ether (v:v = 2:1) preparative chromatography on silica gel. ^1^H NMR (400 MHz, CDCl_3_) *δ* 7.65 (d, J = 7.9 Hz, 2H), 7.25–7.17 (m, 4H), 7.13 (d, J = 8.4 Hz, 2H), 5.96 (s, 2H), 2.53 (s, 6H), 2.38 (s, 3H), 1.25 (s, 6H). ^13^C NMR (101 MHz, CDCl_3_) *δ* 155.71, 144.16, 142.77, 140.52, 137.52, 135.27, 132.08, 131.38, 129.59, 129.12, 127.42, 122.31, 121.32, 21.55, 14.58, and 14.45.

ER-Zn: 3 (0.73 mmol, 360 mg) and TPEA-CHO (0.73 mmol, 317 mg) were weighed and added into a 50 ml three-necked flask. Then the activated molecular sieve was added into the bottle with a water separator. Pumped the pressure of the system to vacuum. Under the protection of nitrogen, 10 ml toluene, a drop of acetic acid and a drop of pyridine were added and refluxed overnight. The reaction process was monitored by TLC until the reaction finished. The reaction was further processed after cooling to room temperature. The saturated NaCl solution was used to extract for three times, and the organic phase was selected to remove water by Na_2_SO_4_. After the solid was removed by filtration, the crude product in the filtrate was collected by rotary evaporation. The target product in blue-black solid form (532 mg, 80.1%) was obtained by preparative chromatography with dichloromethane/methanol (v:v, 100:1→90:10) system on silica gel. ^1^H NMR (400 MHz, CDCl_3_) *δ* 8.57 (dd, J = 5.1, 1.6 Hz, 2H), 7.66–7.63 (m, 4H), 7.61 (d, J = 1.8 Hz, 1H), 7.42 (d, J = 7.7 Hz, 4H), 7.38 (d, J = 8.3 Hz, 2H), 7.24–7.20 (m, 3H), 7.20–7.16 (m, 5H), 7.12 (dd, J = 8.7, 2.4 Hz, 3H), 6.55 (d, J = 6 Hz, 2H), 6.52 (s, 1H), 5.93 (s, 1H), 3.93 (s, 4H), 3.20 (t, J = 5.8 Hz, 2H), 2.91 (t, J = 5.5 Hz, 2H), 2.56 (s, 3H), 2.37 (s, 3H), 2.11 (s, 3H), 1.28 (s, 3H), and 1.24 (s, 3H). ^13^C NMR (101 MHz, CDCl_3_) *δ* 174.77, 158.46, 155.07, 152.89, 149.77, 149.26, 148.83, 143.97, 142.53, 140.30, 140.03, 138.28, 137.63, 137.56, 137.25, 136.92, 135.43, 133.06, 132.34, 131.08, 129.52, 129.47, 127.40, 125.20, 123.52, 122.46, 122.20, 120.46, 117.82, 113.97, 112.62, 106.55, 60.04, 52.66, 40.91, 21.54, 21.01, 14.79, 14.58, and 14.33. HRMS (positive mode, m/z): Calcd: 913.39896, found: 913.39630 for [M + H]^+^ and Calcd: 935.38090, found: 935.37921 for [M + Na]^+^.

### 2.3 Spectral Characterization

7.78 mg ER-Zn was accurately weighed and then dissolved with 8.52 ml DMSO to prepare 1 mM ER-Zn storage solution. No special instructions, the test system is 50 mM HEPES solution with 60% DMSO (containing 100 mM KNO_3_, pH = 7.2).

#### 2.3.1 Absorption and Excitation Spectra Tests

10 μM ER-Zn solution was prepared with 3 ml buffer solution, and its absorption and fluorescence spectra were collected. Then, 1 μl ZnCl_2_ (3 mM) was equivalently dropped into the test system to record the absorption titration spectra, fluorescence titration spectra, and excitation titration spectra. The absorption band was collected from 480 to 750 nm, the excitation spectra test parameters are as follows: the emission at 700 nm and excitation collection band from 475 to 685 nm, the slit is 4 * 4 nm.

#### 2.3.2 Selectivity and pH Dependence Assays

Selectivity test: The different metal ions 2 mM Mg^2+^, K^+^, Ca^2+^ and Na^+^, and 10 μM Ni^2+^, Co^2+^, Al^3+^, Cr^3+^, Cu^2+^, Fe^3+^, Mn^2+^, Pb^2+^ solutions, respectively, were dripped to the probe solution before collecting their excitation spectra. Then, 10 μl ZnCl_2_ solution (3 mM) was added into the test system containing different ions to determine their excitation spectra.

pH-dependent experiment: Using NaOH and HCl, the buffer solution (50 mM HEPES, containing 60% DMSO, 100 mM KNO_3_, pH = 7.2) was adjusted to pH range of 2–10, then 30 μl ER-Zn storage solution (1 mM) was added to the solutions to make the final concentration as 10 μM. Finally, 10 μl ZnCl_2_ storage solution (3 mM) was dropped into the above solutions, and the fluorescence spectra were performed.

### 2.4 Cell Imaging

#### 2.4.1 Cell Culture

The HeLa cells used in the experiment were cultured in the air with 5% CO_2_ at 37°C. The cells were cultured in Dulbecco’s Modified Eagle Medium (DMEM, Invitrogen) containing 10% fetal bovine serum (FBS), penicillin (100 units/ml), and streptomycin (100 mg/ml). There is no special indication that HeLa cells are used for cell imaging.

#### 2.4.2 Cytotoxicity Experiment

MTT (3-(4,5-dimethylthiazol-2)-2,5-diphenyltetrazolium bromide) method was used to determine the number of living cells by using MTT to produce purple precipitate formazan with the mitochondrial succinate dehydrogenase of living cells. HeLa cells in the exponential growth phase were collected and inoculated into 96-well plates. About 5,000 cells and 100 μl medium containing 10% FBS were added to each plate. HeLa cells in 96-well plates were placed in a 37°C, 5% CO_2_ incubator. After cell adherent growth for 12 h, the original culture medium was washed away, and 200 μl ER-BDP with different concentrations were added to each well, and then put back into the incubator for 24 h. Then 30 μl MTT solution was added to each well. After incubation in the incubator for 12 h, the supernatant was carefully removed, and 200 μl DMSO was added to each well to fully dissolve the purple precipitate. Finally, the absorbance of each well at 490 nm was measured by a microplate reader. Each group of experiments was measured three times in parallel.

#### 2.4.3 Colocalization Experiment

Two groups of cells were prepared, and the co-localization experiments between the nucleus, ER and ER-Zn were carried out respectively. Two groups of cells were treated with 100 nM ER-Tracker™ Blue-White DPX dye for 30 min and 100 nM DAPI for 10 min, respectively. The cells were taken out and washed three times with PBS buffer, then incubated with 5 μM ER-Zn solution for 30 min and washed three times with PBS buffer.

The excitation wavelength and emission channel of ER-Blue Tracker dye were as follows: the excitation wavelength was 405 nm, and the emission channel was 415–450 nm; the excitation wavelength of the probe ER-Zn was 570 nm, and the emission channel was 660–720 nm. For DAPI, the excitation wavelength was 405 nm, and the emission channel was 430–500 nm; the excitation wavelength of ER-Zn was 633 nm, and the emission channel was 645–700 nm.

#### 2.4.4 Detection of Exogenous Zn^2+^

The cultured cells were washed three times with metal-free PBS solution and then incubated with 5 μM ER-BDP solution at room temperature for 2 h. Remove the probe solution, wash the cells three times with PBS solution without metal ions, and then observe them with a laser confocal fluorescence microscope. The introduction of exogenous zinc in cells was carried out by incubation in a 1:1 mixture of 5 mM ZnCl_2_ and 5 mM 2-mercaptopyidine-N-oxide aqueous solution (diluted to a specific concentration by DMEM). After incubation for 20 min, the imaging was performed with a laser confocal fluorescence microscope. After imaging, the cells were washed three times with PBS without metal ions and then treated with 50 μM TPEN (diluted by the TPEN storage solution through DMEM medium) for 20 min, and then washed once with PBS.

#### 2.4.5 Detection of Endogenous Zn^2+^ Under Endoplasmic Reticulum Stress

Time-course imaging: The cells were incubated with 5 μM ER-Zn for 2 h and then washed with PBS buffer three times. Then, the cells were incubated with 25 μg/ml thapsigargin (TG) and collected images with time.

Cell imaging under different stimuli: Three groups of cells were stimulated by TM and TG to induce ER stress, respectively. The cells were treated with medium, 25 μg/ml tunicamycin (TM), and 25 μg/ml TG for 12 h, respectively. After incubation with 5 μM ER-Zn solution for 2 h, the cells were washed three times with PBS buffer. The cells were washed with PBS solution three times, and the cells were imaged.

## 3 Result and Discussion

### 3.1 Rational Design and Spectral Characterization of ER-Zn

**FIGURE 1:**
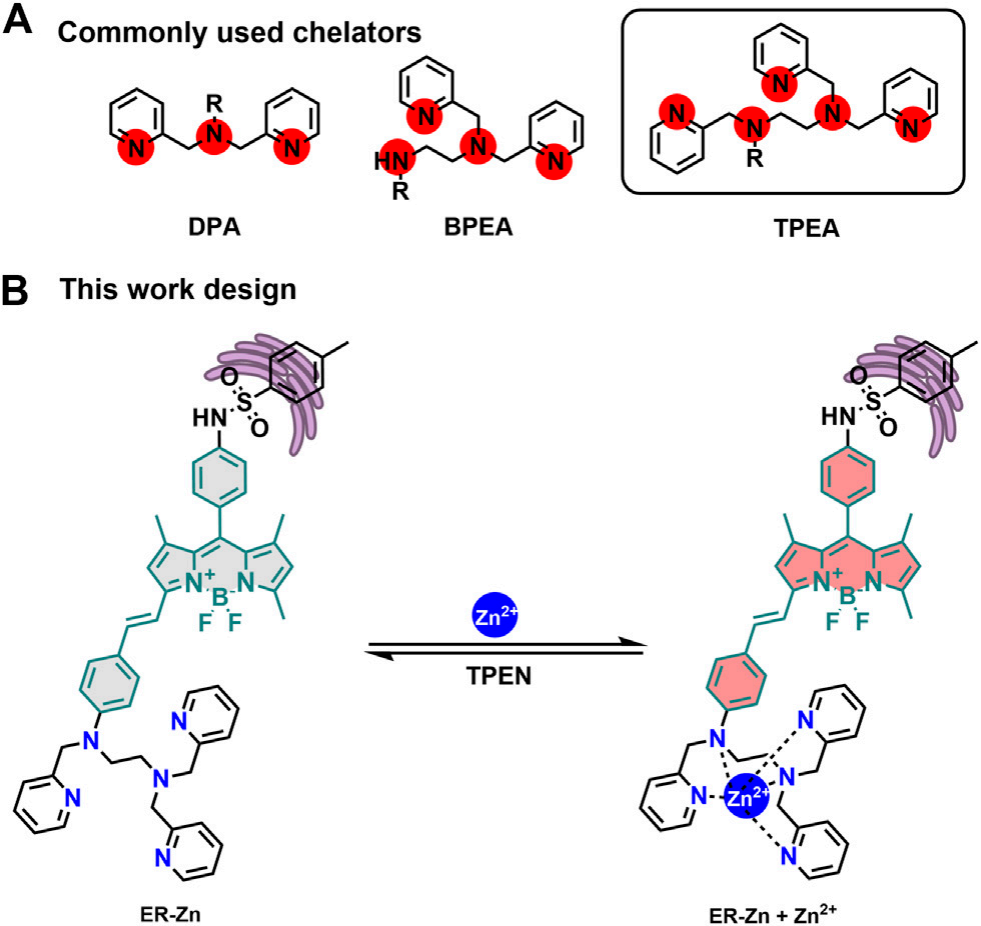
**(A)** Commonly used Zn^2+^ chelators. **(B)** The Zn^2+^ sensing mechanism of ER-Zn.

The design of Zn^2+^ fluorescent probes has encountered the following problems so far: 1) Sensitivity and selectivity. Since DPA (Di (2-methylpyridine) amine) was first linked with fluorescein, the chelating group has become the most commonly used recognition group for the construction of Zn^2+^ sensors (Walkup et al., 2000). However, binding ability and stability are still the main problems for high sensitivity monitoring of Zn^2+^ in biological systems. Hence, we improved the binding ability, coordination rate, and zinc complex stability by increasing coordination sites to form DPA derivatives with five ligands as TPEA (Figure 1A). 2) Organelle-targeting capability. To achieve the ER targeting effect, glibenclamide fragment acting on K^+^ channel in the ER was selected as the targeting group of the probe (Zünkler et al., 2004). 3) Imaging depth limitation. We expanded the conjugation of the BODIPY fluorophore to achieve depth imaging in biological tissues. In addition, the conjugated structure has an obvious ICT effect and can achieve quantitative imaging. To this end, we constructed a highly selective NIR ER targeting ratiometric Zn^2+^ probe, ER-Zn (Figure 1B). Its synthetic route and structural characterization including NMR and MS are shown in Supplementary Figures S1–S4.

**FIGURE 2:**
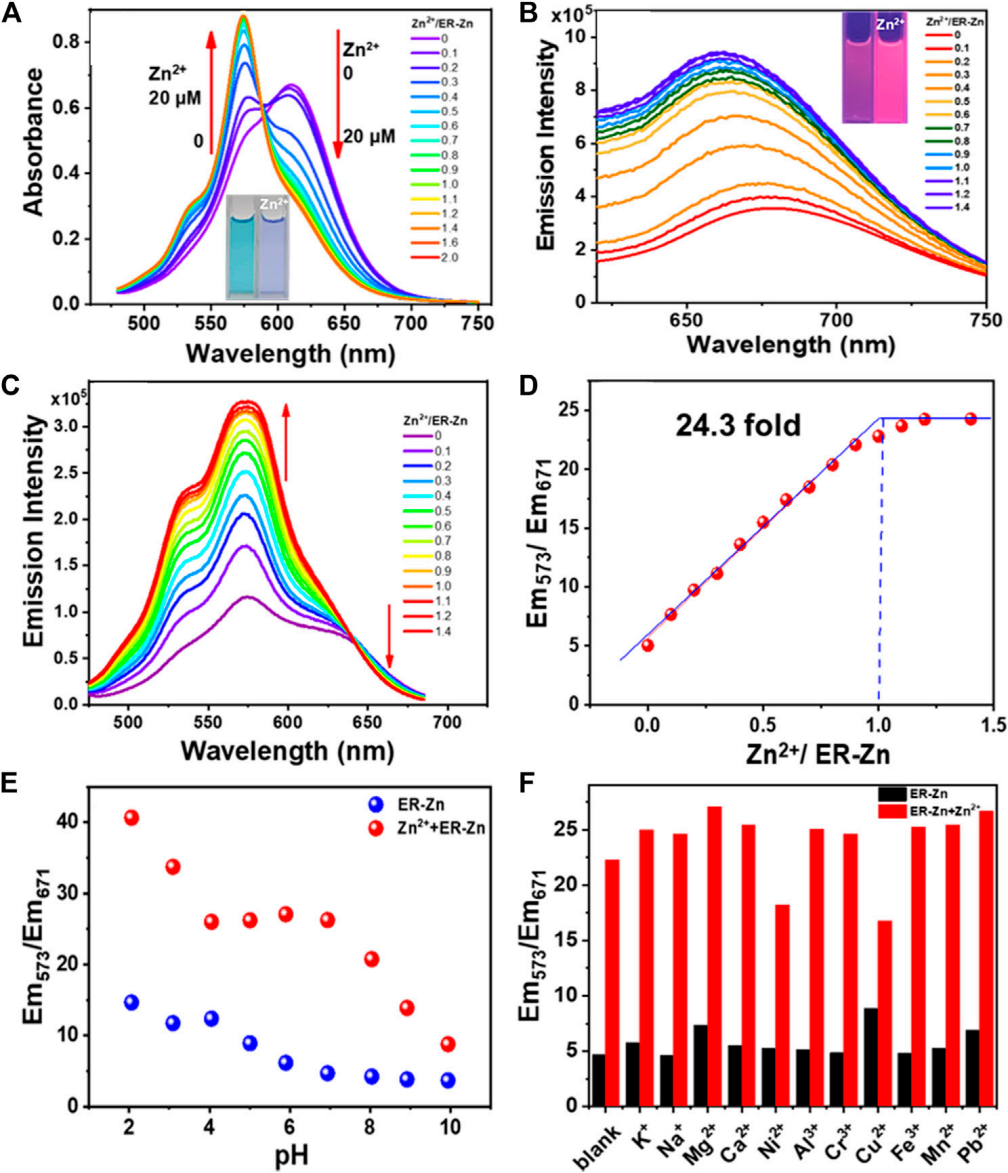
**(A)** Absorption titration spectra of 10 μM ER-Zn with Zn^2+^. The inset: photographs of 10 μM ER-Zn with 0 and 1.0 eq. Zn^2+^ under the natural light; **(B)** Zn^2+^ fluorescence titration spectra of 10 μM ER-Zn with Zn^2+^. The inset: photographs of 10 μM ER-Zn with 0 and 1.0 eq. Zn^2+^ under the 365 nm UV light. λ_ex_ = 600 nm; **(C)** Excitation spectra of 10 μM ER-Zn upon the addition of Zn^2+^. **(D)** Scatter plot between Em_573_/Em_671_ with different concentrations of Zn^2+^. λ_em_ = 700 nm, the excitation collection band is 475–685 nm, the slit is 4 * 4 nm; **(E)** Em_573_/Em_671_ of ER-Zn and ER-Zn + Zn^2+^ in the pH range from 2 to 10; **(F)** Selectivity of ER-Zn and ER-Zn + Zn^2+^ under different cation conditions.

The probe molecule ER-Zn was prepared as a 10 μM solution in HEPES buffer (50 mM, containing 60% DMSO, 100 mM KNO_3_, pH = 7.2). Absorption and fluorescence titration were performed by adding different concentrations of Zn^2+^. As can be seen from Figure 2A, with the increase of Zn^2+^ concentration, the absorbance at 610 nm decreases continuously until it disappears, while a new absorption peak at 575 nm appears and keeps increasing. Therefore, when Zn^2+^ was added, the absorption peak was blue-shifted from 610 to 575 nm with an isosbestic point appearing at 588 nm, and the solution color changed from cyan to blue. The nitrogen atom in the chelating group of the free probe had a strong electron donating ability. However, Zn^2+^ binding TPEA weakened the electron-donating ability of nitrogen atom, which diminished the ICT effect of the structure, inducing a hypochromatic shift of absorption peak.

Subsequently, the Zn^2+^ fluorescence titration spectra of ER-Zn were determined. As can be seen from Figure 2B, with the increase of Zn^2+^ concentration, the emission intensity at 660 nm was enhanced. Based on this, we further tested its excitation spectra with a collection band of 475–685 nm at 700 nm emission. The excitation titration spectra of ER-Zn upon Zn^2+^ were shown in Figure 2C, with the addition of Zn^2+^, the fluorescence intensity at 700 nm excited with 573 nm increased, meanwhile, the fluorescence intensity at 700 nm excited with 671 nm decreased. This excitation ratio sensing ability enables ER-Zn to present dual-excitation with single-channel emission ratiometric imaging, which means that during the titration process of Zn^2+^, the emission at 700 nm under 573 nm excitation enhances, while the emission at 700 nm under 671 nm excitation underwent slightly decrement. The emission ratio (Em_573_/Em_671_) reached equilibrium when Zn^2+^ was added to 1 eq. It indicated that ER-Zn binds with Zn^2+^ in the manner of 1:1. In addition, the detection limit (3σ/slope) of this probe was estimated to be 31.8 nM (Figure 2D). It was demonstrated that ER-Zn and Zn^2+^ exhibited the characteristic of ratiometric sensing, which can eliminate background fluorescence, providing a basis for the realization of quantitative detection in the cell.

### 3.2 The Associate Mechanism of ER-Zn to Zn^2+^

According to the Zn^2+^ fluorescence titration spectra of ER-Zn, when the concentration of ER-Zn and Zn^2+^ was 1:1, the ratio of Em_573_/Em_671_ reached plague, indicating that ER-Zn associated with Zn^2+^ was 1:1. To further determine the binding ratio of ER-Zn to Zn^2+^, the work curve was determined as the concentration ratio (ER-Zn/ER-Zn + Zn^2+^) ranges from 0.1 to 1. As shown in Supplementary Figure S5, when the concentration ratio was 0.5, the fluorescence intensity at 660 nm came up to maximum, that is, the binding ratio of ER-Zn and Zn^2+^ is 1:1. Notably, the dissociation constant of the ER-Zn-Zn^2+^ complex was calculated as 3.65 nM (Supplementary Figure S6). In addition, HRMS determination of the probe solution in the presence of Zn^2+^ was also carried out (Supplementary Figure S7). We can find that the mass peak of [ER-Zn + Zn]^2+^ is 488.1596, which is consistent with the predicted peak of 488.1599, further proving that the association ratio of ER-Zn to Zn^2+^ is 1:1.

### 3.3 pH Dependence and Selectivity of ER-Zn

After confirming the ratiometric sensing ability of ER-Zn to Zn^2+^, we further investigated the pH stability and sensing specificity of ER-Zn. Firstly, we examined whether the response behavior of ER-Zn to Zn^2+^ would be interfered with the physiological pH range. As shown in Figure 2E, the Em_573_/Em_671_ ratio of ER-Zn in the absence and presence of Zn^2+^ did not change significantly in the pH range of 4–8. It can be inferred that ER-Zn can be applied to detecting the dynamic change of Zn^2+^ in the physiological environment.

Next, we explored the selectivity and anti-interference ability of ER-Zn to Zn^2+^. It can be seen from Figure 2F that Em_573_/Em_671_ of ER-Zn added with different metal ions was similar to that of the blank sample, indicating ER-Zn’s excellent selectivity. Notably, the solution containing different metal ions was then added with equivalent Zn^2+^, it was found that Em_573_/Em_671_ exhibited a significant enhancement compared with that of without the addition of Zn^2+^, only the solution containing Ni^2+^ and Cu^2+^ increased slightly. The content of Ni^2+^ and Cu^2+^ in ER and even cells are much less than that of Zn^2+^, so their influence on ER-Zn recognition of Zn^2+^ can be ignored. The above result revealed that ER-Zn has a specific response to Zn^2+^ at physiological pH, which is expected to be used to detect the Zn^2+^ level change in living cells.

### 3.4 Reversibility of ER-Zn

Reversible responses are very important to studying the dynamic changes of Zn^2+^ level in cells. To this end, the reversibility of ER-Zn with between Zn^2+^ and TPEN was studied. Supplementary Figure S8 showed that Em_573_/Em_671_ ratio increased after the Zn^2+^ addition, and the TPEN addition made Em_573_/Em_671_ ratio decreased. The reversibility cycle can be repeated at least four times, providing a clue for the study of Zn^2+^ dynamic change in the cell.

### 3.5 Photostability and Colocalization of ER-Zn in Cells

To study the cytocompatibility of ER-Zn and select the appropriate concentration for subsequent cell experiments, MTT was used to determine the cell survival rate after the HeLa cells were incubated with ER-Zn at the concentrations of 0, 5, 7.5, 10, 12.5, 15, 17.5, 20, 22.5, and 25 μM for 24 h. When the concentration of ER-Zn reached 25 μM, the cell survival rate was even close to 100% (Supplementary Figure S9). Therefore, ER-Zn has good biocompatibility and can be used for bioimaging. Considering the good photophysical properties of ER-Zn, 5 μM can be selected as the following cell imaging experiment.

Then we studied the photostability of ER-Zn in cells. The cells were incubated with ER-Zn for 2 h and were imaged every 2 min after continuous illumination. As can be seen from Supplementary Figure S10, the fluorescence intensity of the green channel and the red channel did not change significantly within 10 min, and the fluorescence ratio remained almost the same, indicating that ER-Zn had good photostability to be suitable for long time dynamic imaging.

Next, to confirm the distribution of ER-Zn in the cell, we conducted a colocalization experiment using ER commercial dye and ER-Zn, and found that the probe and ER commercial dye has a very high overlap, the calculation of Pearson’s colocalization coefficient can reach 0.93 (Supplementary Figure S11). However, its colocalization coefficient with the nucleus is only 0.13. It demonstrated that ER-Zn is mainly located in ER, which is consistent with our original design purpose.

### 3.6 Reversible Cell Imaging of Exogenous Zn^2+^ by ER-Zn

**FIGURE 3:**
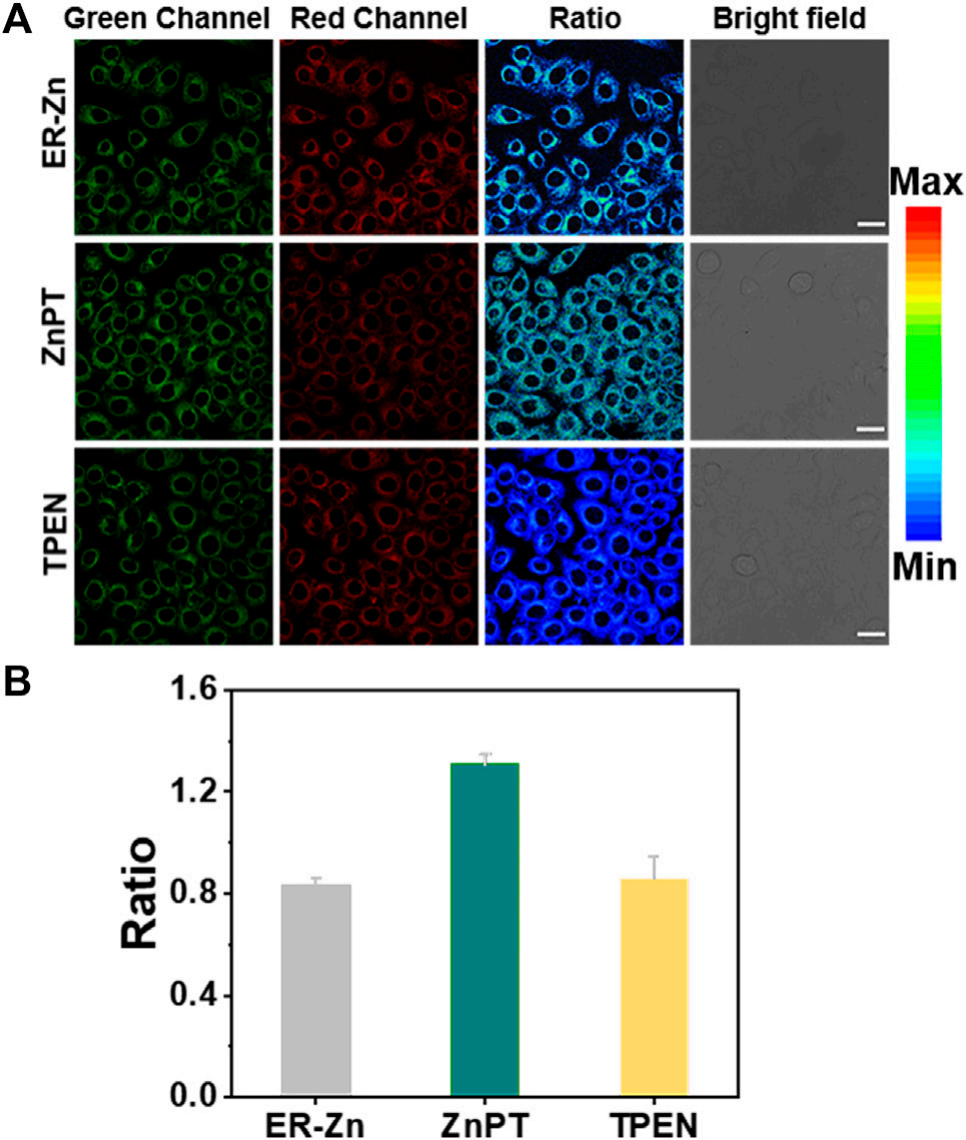
Reversible ratiometric imaging of ER-Zn for exogenous Zn^2+^. **(A)** Ratiometric imaging of the ER-Zn-stained HeLa cells (5 μM, 2 h, 37°C) upon incubation with 5 μM ZnPT for 20 min and subsequent TPEN (50 μM, 30 min) treatment; **(B)** Fluorescence ratio quantification of **(A)**. The corresponding histograms of the determined average fluorescence intensity in cells from *n* = 3 biologically independent experiments, mean ± SD. The green channel: λ_ex_ = 570 nm, λ_em_ = 660–720 nm, the red channel: λ_ex_ = 650 nm, λ_em_ = 660–720 nm, Ratio = F_G_/F_R_, scale bar: 25 μm.

The ER targeting ability of ER-Zn has been confirmed via a colocalization experiment. To further study the sensing ability of ER-Zn on Zn^2+^ in ER, we next conducted cell imaging experiments of endogenous and exogenous Zn^2+^ using ER-Zn. We selected membrane permeable Zn^2+^ carrier (zinc pyrithione, ZnPT) as an exogenous Zn^2+^ supplement and TPEN as an intracellular Zn^2+^ chelator. When the cells were incubated with ER-Zn for 2 h, excitation ratiometric imaging was performed, and the probe showed dual-channel imaging in the cells. Then the cells were incubated with 5 μM ZnPT for 10 min for cell imaging. As shown in Figure 3, the fluorescence intensity of the green channel was significantly enhanced compared with the control group, while the fluorescence intensity of the red channel weakened, and the image ratio was significantly enhanced, showing the elevated Zn^2+^ in cells. When the cells were incubated with 50 μM TPEN for 20 min, the image ratio was significantly decreased. It is proved from the result that ER-Zn can be used for quantitative imaging of exogenous Zn^2+^ in cells, which provides a basis for quantitative and dynamic imaging of endogenous Zn^2+^ in ER.

### 3.7 Zn^2+^ Change Under Endoplasmic Reticulum Stress Regulation

**FIGURE 4:**
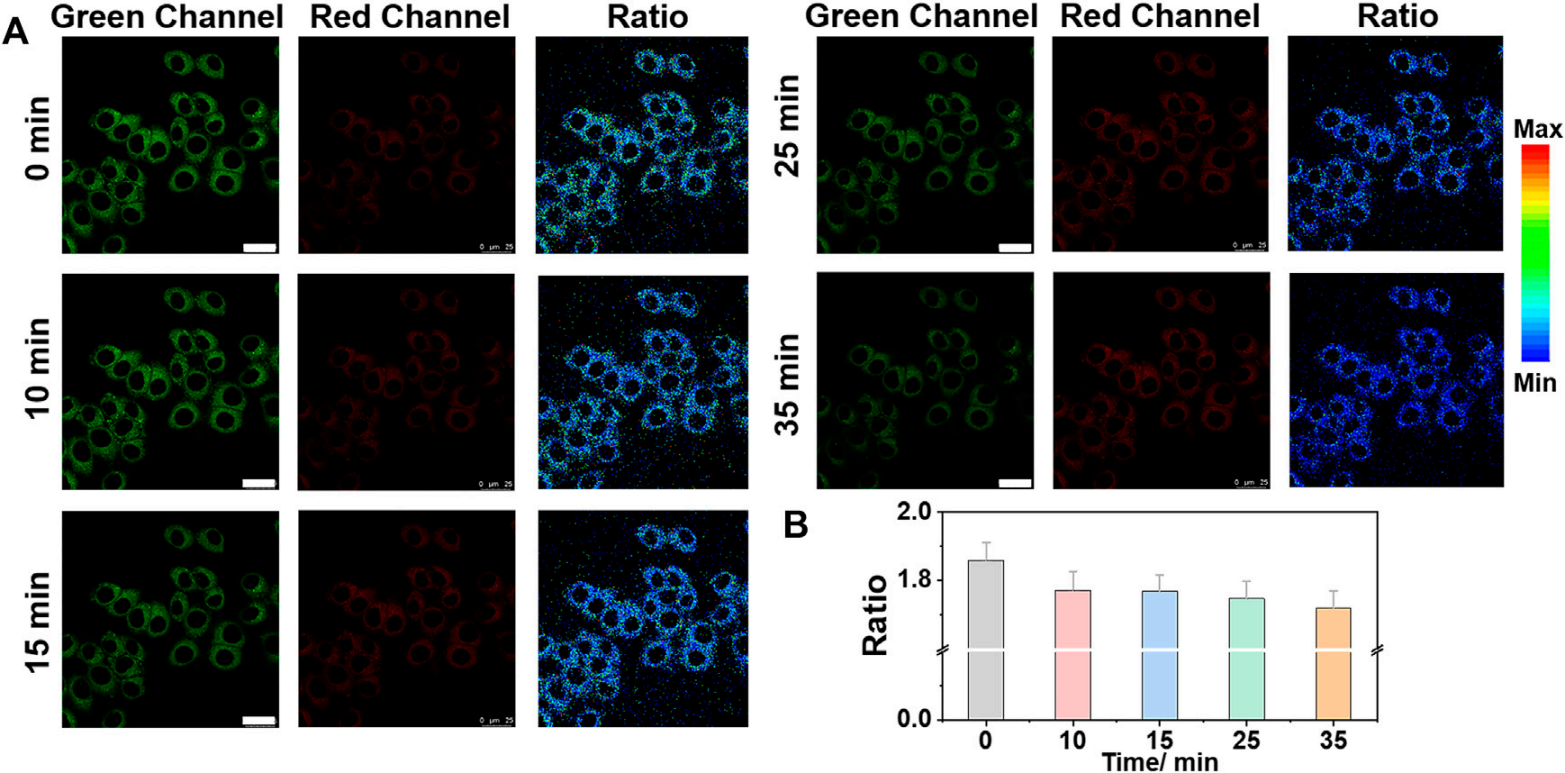
Ratiometric imaging of Zn^2+^ in ER under TG (thapsigargin) stimulation with time. **(A)** Ratiometric images and **(B)** the corresponding fluorescence intensity changes of ER-Zn (5 μM, 2 h) in HeLa cells at ER stress state induced by TG (25 μg/ml) for different time. The green channels: λ_ex_ = 570 nm, λ_em_ = 660–720 nm, the red fluorescence channels: λ_ex_ = 650 nm, λ_em_ = 660–720 nm; Ratio = F_G_/F_R_, Mean ± SD, scale bar: 25 μm.

To study the effect of different stimuli (e.g., TG and TM, etc.) on Zn^2+^ in the ER, we set up cell imaging of endogenous Zn^2+^ by ER-Zn under ER stress. TG can affect ER calcium (Ca^2+^) homeostasis by specific inhibition of ER Ca^2+^-ATPase and induce ER stress (Zhang et al., 2014; Sehgal et al., 2017). Therefore, we selected TG as the agent to stimulate ER stress. We investigated the Zn^2+^ level change in ER of HeLa cells after TG stimulation for different time periods. Figure 4 showed that with the extension of stimulation time, the fluorescence intensity of the green channel gradually decreased and that of the red channel increased slightly, showing that the ER stress-induced by TG can lead to a decreased Zn^2+^ level in ER.

**FIGURE 5:**
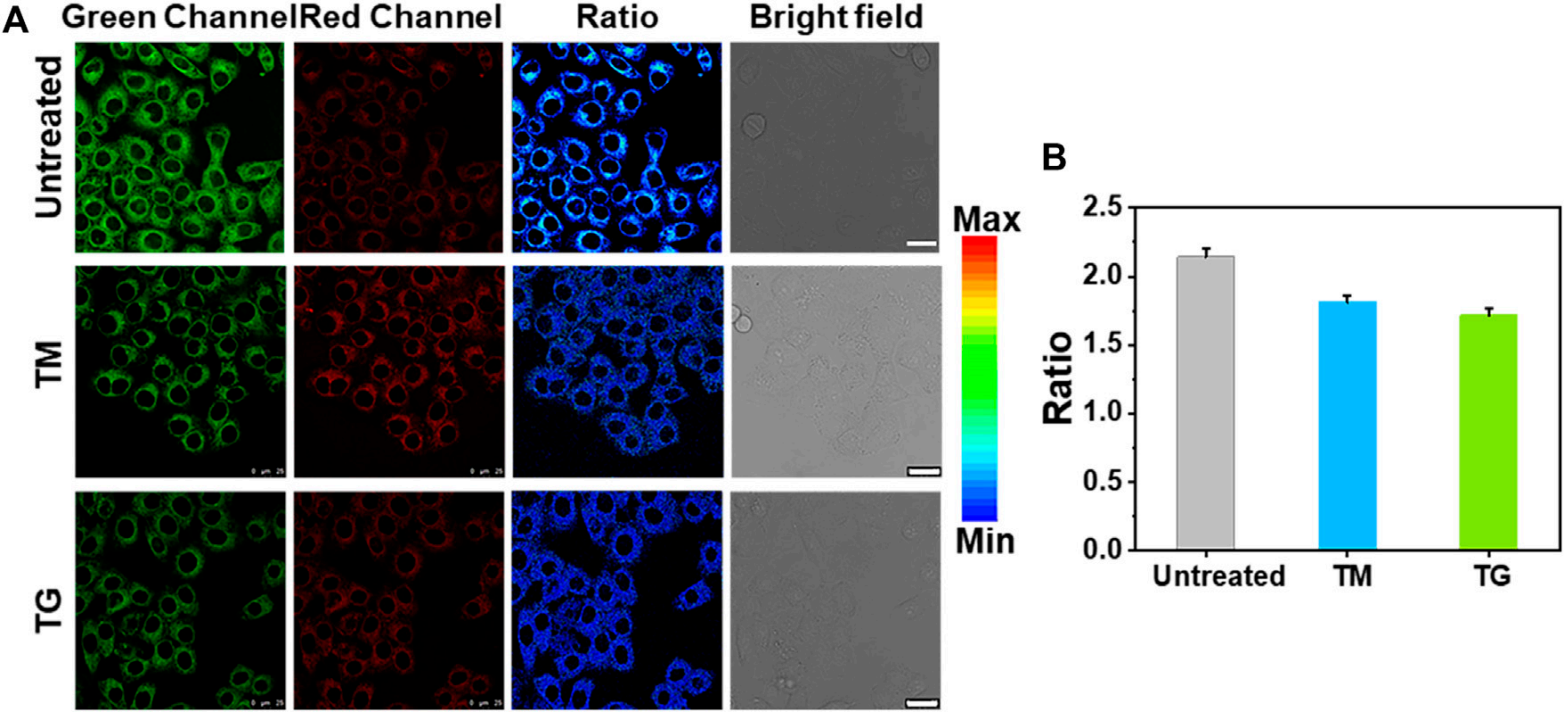
Ratiometric imaging of Zn^2+^ in ER under ER stress regulation by different stimuli. **(A)** Ratiometric images of HeLa cells treated with medium, 25 μg/ml TM and 25 μg/ml TG for 12 h, respectively, then incubatied with ER-Zn (5 μM, 2 h). **(B)** The corresponding mean fluorescence intensity ratio of **(A)**. The green channels: λ_ex_ = 570 nm, λ_em_ = 660–720 nm, the red fluorescence channels: λ_ex_ = 650 nm, λ_em_ = 660–720 nm; Ratio = F_G_/F_R_, Mean ± SD, scale bar: 25 μm.

In addition, TM is also commonly used to induce ER stress (Jackisch et al., 2020; Suganya et al., 2014). We stimulated the cells with TM and TG to produce ER stress. As can be seen from Figure 5, the fluorescence ratio of the cell induced by both TM and TG decreased to varying degrees, further indicating that ER stress regulates the decline of Zn^2+^ level in ER. We speculate that Zn^2+^ homeostasis, like Ca^2+^ homeostasis, is controlled by IP3Rs, which may allow Zn^2+^ to enter the cytoplasm and pump Zn^2+^ from the cytoplasm into ER through TG sensitive ATPase activity (Stork and Li, 2010; Liang et al., 2016). When ER stress occurs, especially after the stimulation of TG and TM, Zn^2+^ will be released from the ER into the cytoplasm, resulting in the decrease of Zn^2+^ in the ER.

## 4 Conclusion

In summary, based on the ICT mechanism, we designed and synthesized a novel NIR ratiometric fluorescent probe targeting ER, ER-Zn, which has a good ratio response to Zn^2+^, good selectivity, and anti-interference ability. In addition, ER-Zn not only possesses double excitation ratio detection of exogenous Zn^2+^ but made clear the relationship between ER stress regulation by different stimuli and the endogenous Zn^2+^ level in the ER. ER-Zn with the ability of NIR imaging provides a reliable technical basis and design strategy for the further development of quantitative imaging of Zn^2+^ probes *in vivo*. We envision that this probe can be applied to screen drugs for diseases related to ER stress regulation.

## Data Availability

The raw data supporting the conclusion of this article will be made available by the authors, without undue reservation.
