# Supplementary material for: An Endoplasmic Reticulum-Targeted Ratiometric Fluorescent Molecule Reveals Zn2+ Micro-Dynamics During Drug-Induced Organelle Ionic Disorder
Source: Front Pharmacol. 2022 Jun 6;13:927609. doi: 10.3389/fphar.2022.927609 (PMC9207263; doi:10.3389/fphar.2022.927609)
Supplement: Supplementary file 1 [file DataSheet1.docx]

Supplementary Material

An endoplasmic reticulum-targeted ratiometric fluorescent molecule reveals Zn^2+^ micro-dynamics during drug-induced organelle ionic disorder

Hongbao Fang, Yaheng Li, Shankun Yao, Shanshan Geng, Yuncong Chen*, Zijian Guo*, Weijiang He*

**Figure S1** Synthesis route of **ER-Zn**

**Figure S2** ^1^H NMR of **ER-Zn** (400 MHz, CDCl_3_)

**Figure S3** ^13^C NMR of **ER-Zn** (101 MHz, CDCl_3_)


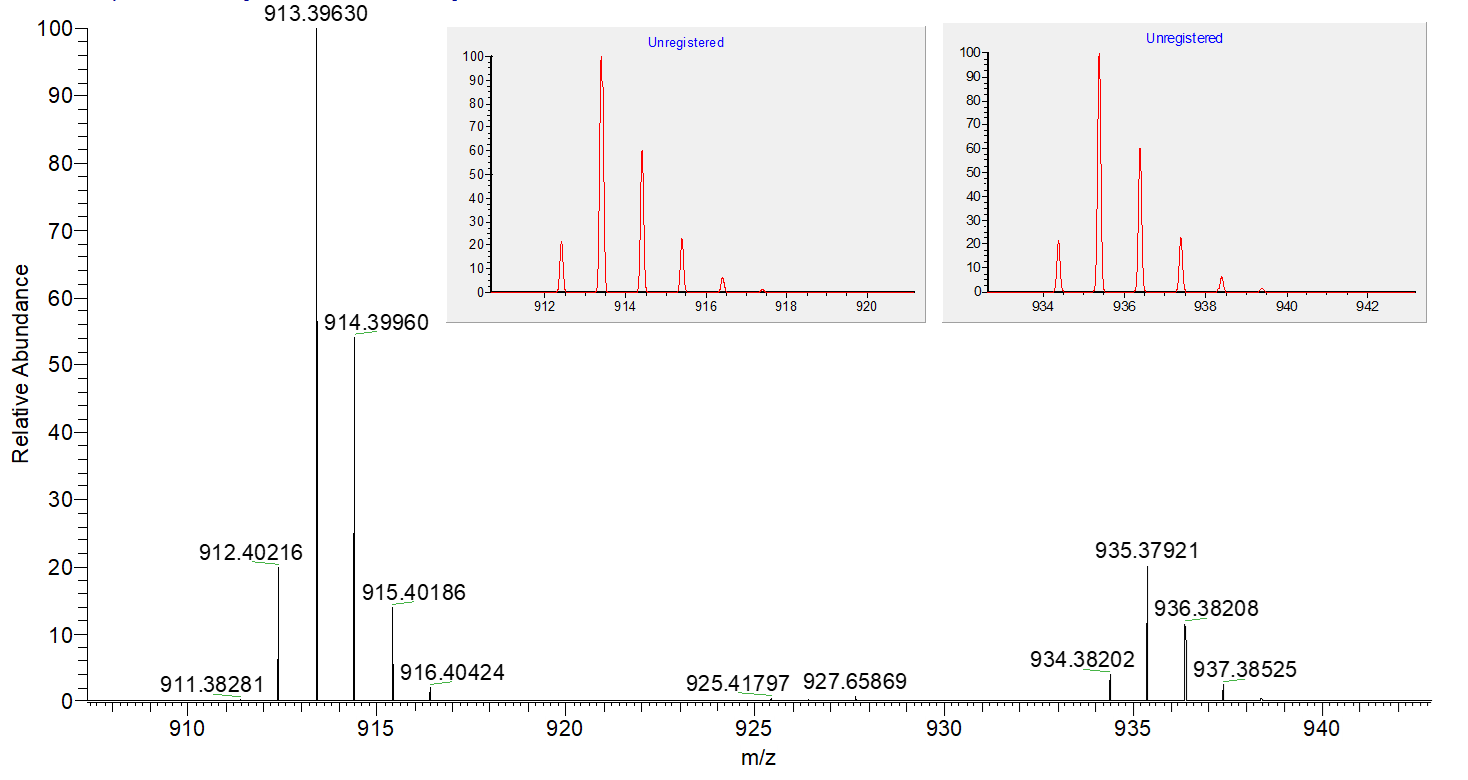


**Figure S4** HRMS of [**ER-Zn+H/Na]^+^**


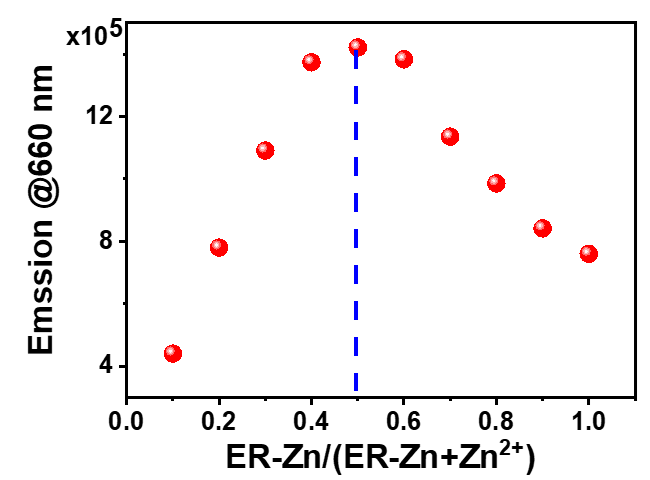


**Figure S5** Work curve of **ER-Zn**

**
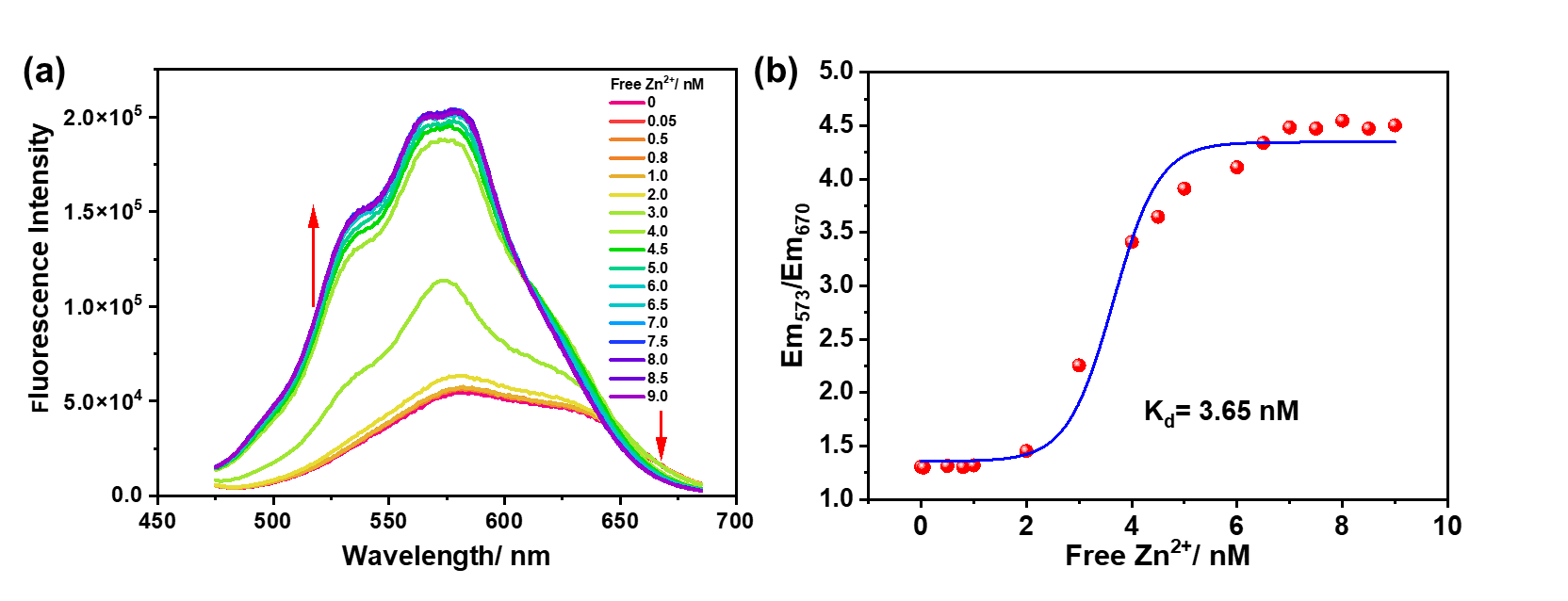
**

**Figure S6** (a) The excitation spectra of **ER-Zn** (10 μM) with the addition of free Zn^2+^ in the HEPES buffer (50 mM, 100 mM KNO_3_, pH 7.2, 10 mM EGTA); (b) Zn^2+^ binding curve based on emission intensity ratio Em_573_/Em_640_ and its fitting for dissociation constant determination.


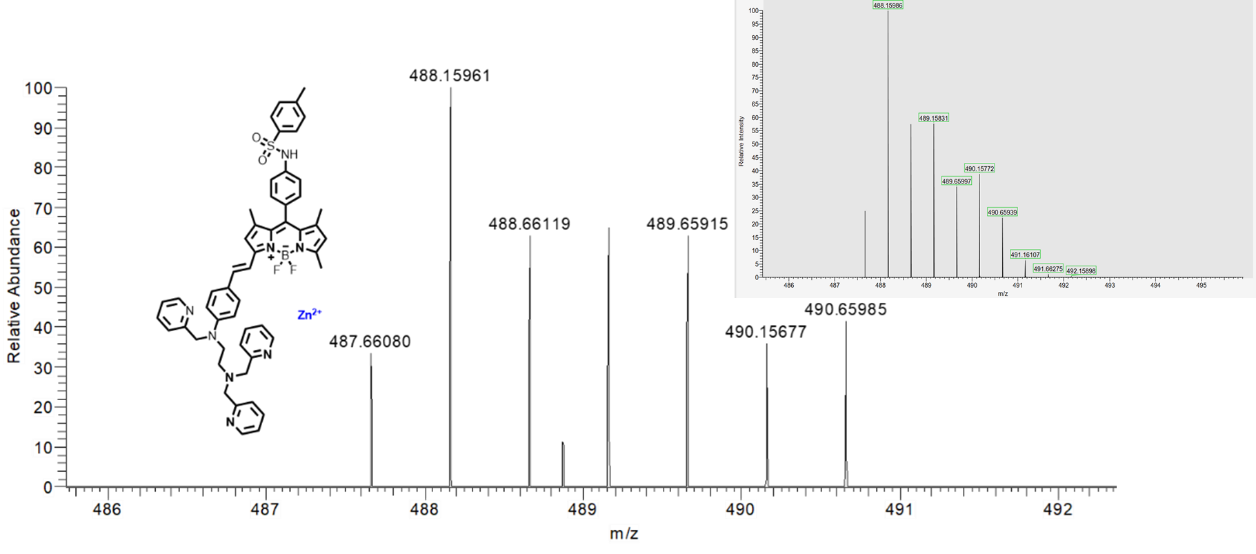


**Figure S7** HRMS of [**ER-Zn**+Zn]^2+^

**Figure S8** Reversibility of 10 μM **ER-Zn** response to Zn^2+^

**Figure S9** Cytotoxicity of **ER-Zn** with different concentrations in HeLa cells.


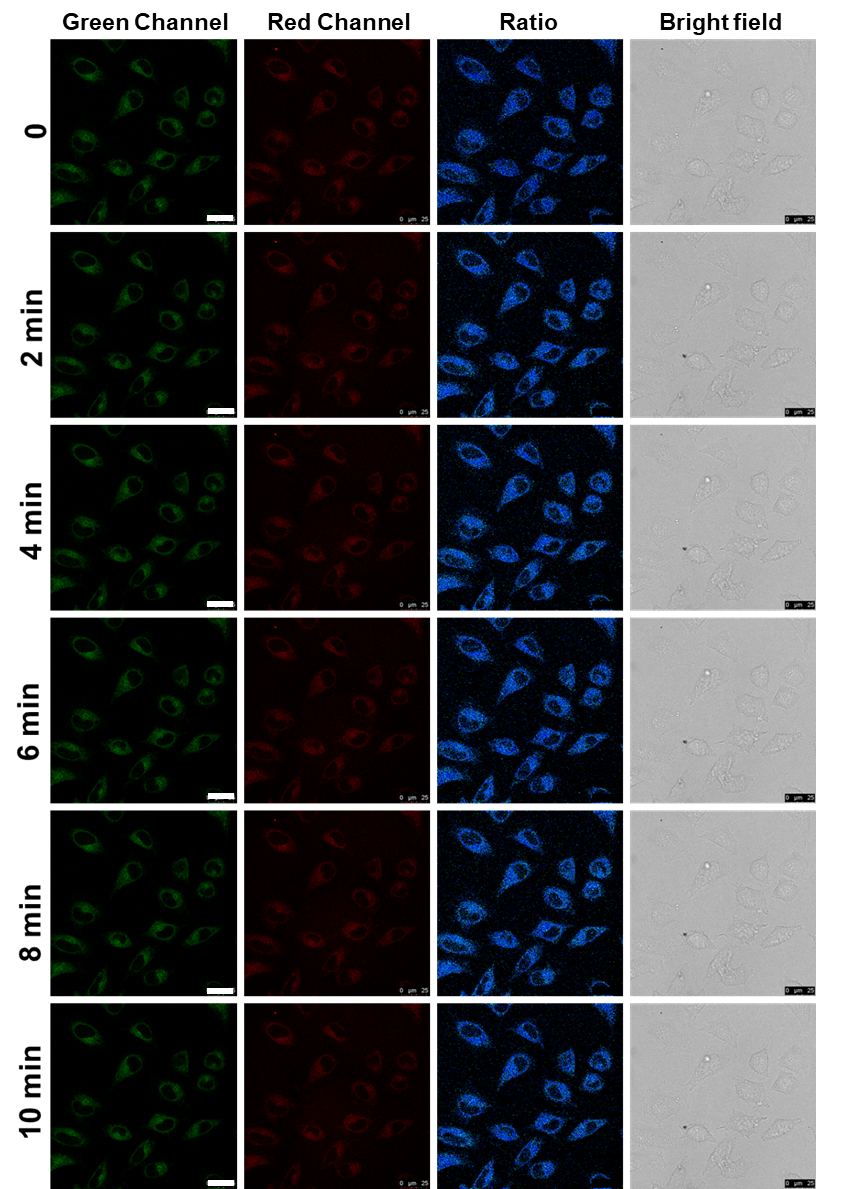


**Figure S10** Photostability experiment of 5 μM **ER-Zn** incubated with cells for 2 h. green channel: λ_ex_ = 570 nm; red channel: λ_ex_= 650 nm, λ_em_= 660-720 nm, scale bar: 25 μm.


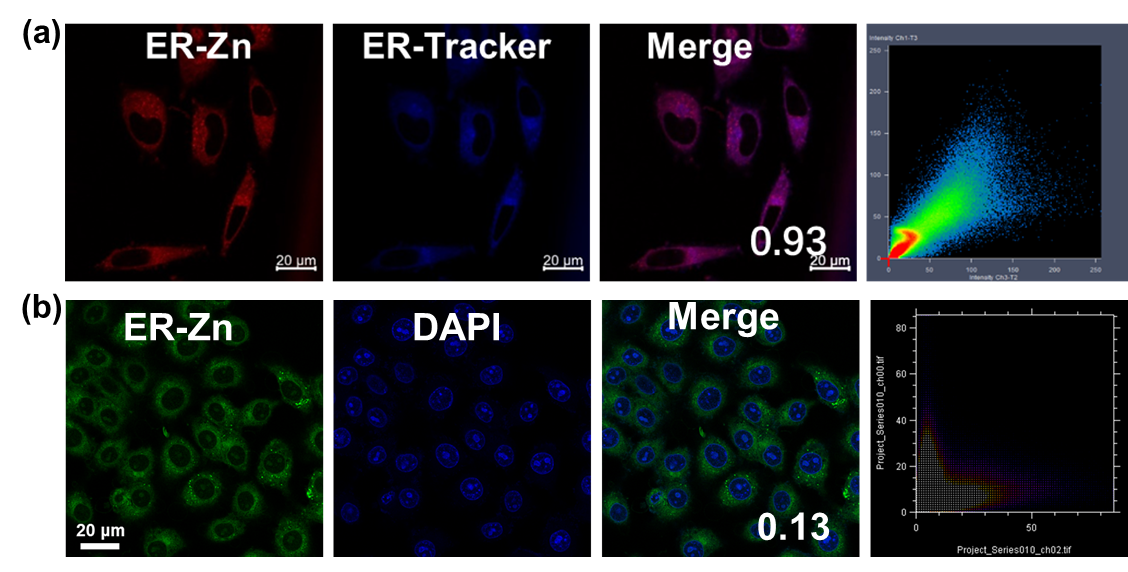


**Figure S11** The colocalization experiment of **ER-Zn** with commercial ER tracker (a) and commercial nuclei tracker(b). Scale bar: 20 μm.
